# Need for Vaccination Policies to Face Asymptomatic Monkeypox Virus Infection

**DOI:** 10.3390/vaccines10122020

**Published:** 2022-11-26

**Authors:** Alexandre Vallée

**Affiliations:** Department of Epidemiology-Data-Biostatistics, Delegation of Clinical Research and Innovation (DRCI), Foch Hospital, 92150 Suresnes, France; al.vallee@hopital-foch.com

The emergence of the monkeypox virus (MPXV) outbreak in 2022 represents a global threat to health [1,2]. Between 1 January and 17 August 2022, a total of 69,000 laboratory-confirmed cases and 25 deaths have been observed by the World Health Organization (WHO) from 106 countries/territories/areas [3]. On 23 July 2022, due to the rapid increase in monkeypox cases, the WHO declared the escalating worldwide monkeypox epidemic a Public Health Emergency of International Concern. Monkeypox is a contagious disease requiring close physical contact with a person carrying the virus or sexual intercourse [4]. MPXV outbreak mainly affects men who have sex with men (MSM) [5].

Researchers have investigated several questions that could explain the extraordinary surge in cases [6]. One of the hypotheses is that a significant proportion of MPXV infections remain undiagnosed or asymptomatic, allowing for unknowing transmission. This possible mode of transmission remains a major challenge. A recent multi-country study showed that three men among 224 men with confirmed serology for MPXV exposure had no symptoms [7]. The presence of asymptomatic MPXV cases shows that the virus may be transmitted in the absence of any symptoms, challenging appropriate public health policies.

However, asymptomatic cases have negligible weight in the spread of orthopox viruses [8]. A recent study has shown that MPXV could be detected in the upper respiratory tract of asymptomatic contacts of MPXV cases [7]. Furthermore, asymptomatic transmission from or to the anorectum in combination to other body sites may play a role. Several case reports have suggested that unprotected intercourse with several sex partners is driving the community spread of this virus [4,9,10,11]. A recent study has shown that MPX DNA was detected in the saliva of all MPXV cases and also in other samples, such as semen, urine, feces, nasopharyngeal swabs, and rectal swabs [12]. Perio-Mestres et al. concluded that infectivity could be observed in all bodily fluids and that research in this area is key to improving the detection of secondary and asymptomatic cases [12]. Thus, contact with breached anogenital mucosal membranes could be an unrecognized transmission mode of MPXV [13].

However, the eradication of the MPXV outbreak is primarily based on identification, quarantining symptomatic cases, and then tracing their contacts [14]. Nevertheless, undiagnosed or asymptomatic cases could have a much more significant role in transmission and outbreak among MSM due to a dense sexual network, including anonymous contacts of several MSM, hampering efficient contact tracing. Thus, unnoticeable symptoms may explain the insufficient results observed in self-isolation at symptom onset to counteract the outbreak. Appropriate awareness and public health vaccination campaigns for high-risk populations should highlight the possible role of asymptomatic transmission through intercourse [8,15].

Some studies have investigated the attitudes and hesitancy to be vaccinated among the general population. All showed an insufficient knowledge of MPX [16,17,18] depending on age, gender, marital status, education, income level, or place of residence [17,19]. A significant part of the population thought that MPXV is spreading more widely among specific groups, for instance, sexual minorities and migrants [15,17,18]. Although there is a high prevalence of MPXV vaccination acceptance in the LGBTQI+ community, vaccine acceptance from healthcare workers and the general population remains low [18]. Among specific populations, such as HIV or PrEP patients, 33.6% declared to be hesitant about MPXV vaccine acceptance [15], and only 44% of participants in another study showed high intention to self-isolate after a diagnosis of MPX [20]. Thus, specific communication highlighting the benefits of the MPXV vaccine should be introduced. Epidemiological patterns are essential to developing efficient vaccination strategies. However, the models used to control the current MPXV outbreak are not entirely appropriate due to the specific age and population targeted by this virus [21]. Nevertheless, several countries have introduced the MPXV vaccine, especially in the US, where the CDC provided age-wise MPXV vaccination information. Moreover, numerous countries deployed many vaccine doses: e.g., 750,000 for Canada, 450,000 for Australia, 250,000 for France, and 150,000 for the UK.

Considering the rapid spread of this infection and the number of people at risk, the amount of available MPXV vaccines remains low. To face all these problems, healthcare workers should be aware of all the transmission routes of MPXV, including a possible asymptomatic spread, and should reinforce the importance of efficient vaccination campaigns.

## Data Availability

Not applicable.
